#  Evaluation of Sorbitol-Methanol Co-Feeding Strategy on Production of Recombinant Human Growth Hormone in *Pichia Pastoris*

**Published:** 2017

**Authors:** Saeed Azadi, Arash Mahboubi, Nasser Naghdi, Roya Solaimanian, Seyyed Alireza Mortazavi

**Affiliations:** a *Pharmaceutical Sciences Research Center, Shahid Beheshti University of Medical Sciences, Tehran, Iran.*; b *Food Safety Research Center, Shahid Beheshti University of Medical Sciences, Tehran, Iran.*; c *Department of Pharmaceutics, School of Pharmacy, Shahid Beheshti University of Medical Sciences, Tehran, Iran.*; d *Department of Physiology and Pharmacology, Pasteur Institute of Iran, Tehran, Iran.*; e *Department of Pharmaceutics, Pharmaceutical Sciences Branch, Islamic Azad University, Tehran, Iran.*

**Keywords:** Fermentation, *Pichia Pastoris*, Mixed-feed strategy, Sorbitol

## Abstract

Recombinant protein production in* Pichia pastoris *is based on alcohol oxidase promoters which are regulated by methanol. However, the use of methanol has several disadvantages, which is why current trends in bioprocess development with *Pichia pastoris (P. pastoris*) are focusing on methanol mixed feeding strategies. This work aimed to develop a new experimental method and compare the effect of various fractions of sorbitol in mixed feeding strategy with stepwise addition of methanol to maximize the productivity of human growth hormone. Accordingly, fed-batch culture performed with a mixed feed of sorbitol/methanol. Sorbitol at concentrations of 30, 40, 50 and 60 gram per liter was added in batch-wise mode to the medium at the beginning of induction phase and the rate of methanol addition was increased stepwise during the first 12 h of production and then kept constant. In order to understand the effects of various co-substrate feeding strategies on *P. pastoris* and its production of hGH, cell mass, dry cell weight, total protein, hGH expression level and hGH concentration were analyzed and the results compared with the basic feeding protocol using one-way analysis of variance (ANOVA). According to the obtained results, sorbitol at a concentration of 50 g/L could significantly increase the production yield. Under such optimal conditions cell biomass was 108 g/L (DCW), total protein was 0.807 g/L, expression level was 83.1% and rhGH concentration was 0.667 g/L following 30 h induction.

## Introduction

The methylotrophic yeast *Pichia pastoris* is a well-established expression host which is often used in the production of protein pharmaceuticals (1-3). It can produce high levels of recombinant proteins using a strong and tightly regulated methanol-inducible alcohol oxidase promoter (*AOX1*) (4, 5). The most widely utilized fermentation process of *P. pastoris* consists of a three steps approach (6). During the first step, cells are cultured in batches using a defined medium with glycerol as the carbon source to rapidly achieve high cell densities. As the *AOX1* promoter is repressed by unlimited growth on glycerol (7, 8) recombinant protein expression is repressed at this stage. During the second step, in order to increase biomass production and de-repress the methanol metabolic machinery, a glycerol-limited fed-batch procedure is initiated. This phase leads to gradual de-repression of the enzymes necessary for the dissimilation of methanol and reduces the time necessary for the cells to adapt to growth on methanol (9). Finally, recombinant protein expression is usually induced by feeding methanol as the sole carbon source. 

A fermentation guideline for both Mut^+^ and Mut^s ^strains of *P. pastoris* is available from Invitrogen (10). Stratton *et al.* devised a protocol for *P. pastoris* high cell-density fermentation, in which detailed procedures are provided (6). Using this protocol, in our laboratory, recombinant human growth hormone (rhGH) was produced with *P. pastoris *Mut^+^ in which *AOX1* promoter was induced with methanol for 30 h.

However, the methanol feed rate profiles described by Stratton *et al.* are fixed and may be inapplicable for those strains whose ability to utilize methanol has changed as a result of the expression of heterologous genes. For a fed-batch fermentation process, the substrate feed rate usually needs to be optimized to maximize productivity. Various methods for such optimization have been reported in other production systems (11).

Methanol can act as both the carbon source and the inducer of the expression of recombinant proteins. However, in the presence of high concentrations of methanol, which has a high substrate affinity for alcohol oxidase, *P. pastoris* tends to intoxicate itself with metabolites (formaldehyde, formic acid) (12). Hence, at high concentrations, methanol inhibits the organism’s growth. Therefore, in order to keep methanol concentration below the toxic limit, fed-batch operation has been utilized as the standard protocol for recombinant protein production by* P. pastoris *through *AOX1* promoter (13). 

Since accumulation of methanol leads to cytotoxic effects, so methanol mixed feeding strategies have been studied to partially replace or at least complement methanol with other carbon sources such as glycerol (14-16) glucose (17-19) or sorbitol (20-24). Moreover, the use of a less repressing carbon source may result in higher specific production rates, improving overall productivity and eliminating the need for the tight control of residual substrate levels (25). Among them, sorbitol is a widely accepted non-repressive carbon source for *P. pastoris* (26). The advantages of mixed feeds of sorbitol and methanol for the production of different recombinant proteins with *P. pastoris* have been reported (20-25, 27and 28).

As a follow-up to other studies, this work aimed to compare the effect of various concentrations of sorbitol in mixed feeding strategy with stepwise addition of methanol to develop a new experimental method that would maximize the production of hGH in *P. pastoris*. In this study sorbitol was added batch-wise to the medium at the beginning of the induction phase, continuing with methanol feeding for 30 h. In order to understand the effects of various feeding strategies, based on different co-substrate concentrations, on production of hGH in *P. pastoris*, cell density, total protein, hGH expression level and hGH concentration were analyzed and the results compared with the basic protocol of methanol feeding using one-way analysis of variance (ANOVA). 

## Experimental

*Microorganism, inoculum and media preparation*

*P. pastoris* GS 115 strain Mut^+ ^carrying hGH cDNA under the control of *AOX1* which secrets the target protein into the fermentation broth was streaked from glycerol stock onto YPD-agar containing (g/L): yeast extract (10), peptone (20), dextrose (20) and agar (20) and incubated for 48 h at 30 °C (30). A single colony was inoculated into BMGY medium containing 10 g/L yeast extract, 20 g/L peptone, 13.4 g/L YNB, 4 × 10^−5^ g/L biotin, 10 g/L glycerol and 0.1 M potassium phosphate buffer (29) and incubated at 30 °C in a shaker incubator at 150 rpm, until the culture reached an optical density (OD_600_) of 1-2. 

*Fermentation conditions*

The culture obtained was used as inoculum for a 13 L fermenter containing 3 L of basal salts medium which consisted of (g/L): glycerol (40); K_2_SO_4_ (18); MgSO_4_.7H_2_O (14.9); KOH (4.13); CaSO_4_ (0.9) and 27 mL H_3_PO_4_, plus 4.0 mL of a trace metal stock solution that consisted of (g/L): CuSO_4_.5H_2_O (6); KI (0.09); MnSO_4_.H_2_O (3); H_3_BO_3_ (0.02); MoNa_2_O_4_.2H_2_O (0.20); CoCl_2_ (0.5); ZnCl_2_ (20); FeSO_4_·7H_2_O (65); biotin (0.2) and H_2_SO_4_, 5.0 (mL/L) (30). 

The 13 L bioreactor (Infors, Switzerland), had a working volume of 9.0–10.0 L and included temperature, pH, foam, stirring rate, feed inlet rate and dissolved oxygen control systems. Dissolved oxygen (DO) concentration was maintained above 20% air saturation at 400-700 rpm, using air and, when needed, enriching the inlet air with pure oxygen passing through a digital mass flow controller. Temperature was maintained constant at 30.0 ± 0.1 °C throughout the entire bioprocess. In the first two phases, the pH was held at 5.0 ± 0.2 and then in the production phase was lowered to 3.0 ± 0.2. The pH was maintained at the relevant values, by adding 25% ammonia solution. 

*Induction Phase*

According to the basic protocol, the induction phase was performed with methanol feed containing 12 PTM_1_ trace salts per liter of methanol. The feed rate was set to 3.65 mL/h/L initial fermentation volume for six h. Then, the feed rate was doubled to 7.3 mL/h/L initial fermentation volume. After 6 h, feed rate was further increased to 10.9 mL/h/L initial fermentation volume and maintained throughout the remainder of the fermentation up to 30 h.

To conduct a mixed feed strategy, sorbitol at concentrations of 30, 40, 50 and 60 g/L were added to the fermenter in a batch-wise mode and methanol fed-batch was continued based on the basic protocol in which the rate of methanol addition was increased stepwise during the first 12 h of production and then kept constant. To determine the optimal sorbitol concentration for the yeast growth profile and rhGH production, cell density, total protein concentration and expression level of cultures grown using different concentrations of sorbitol were studied.

*Analytical procedures*

*Cell density measurement*

Optical density (OD) of cell suspensions was measured at 600 nm. The wet cell weight (WCW, g/L) was used as a measure of cell density within the bioreactor. A 1.0 mL sample was centrifuged at 13,000 rpm for 5 min. The pellet was weighed and the supernatant was stored at 4 °C for further analysis. The dry cell weight (DCW, g/L) was calculated using the following equation DCW = 0.35 × WCW which was obtained from a calibration curve prepared using 20 samples that were dried for 24 h at 105 °C.

*Determination of total protein:*

Cell suspension was centrifuged at 13,000 rpm for 10 min (Labnet, USA). Since rhGH was secreted into the culture media, the supernatant was used for the determination of total protein concentration by the Bradford assay using a UV/V is spectrophotometer (PerkinElmer, USA), at 595 nm (31). Bovine serum albumin (BSA) was used as the standard protein for this measurement. The total protein concentration of each sample was then estimated from the constructed standard curve.

*SDS–PAGE and densitometric analysis*

To determine the expression level of rhGH, SDS–PAGE was performed in a Mini-Protean^®^ 3 cell gel apparatus (Bio-Rad, USA) according to the method of Laemmli (32). The samples were dissolved in 10 X sample buffer and incubated at 100 °C for 5 min. About 25 μL of each sample was loaded in the gel of 15% resolving and 5% stacking gel with 0.75 mm thickness at a constant voltage of 100 V. After running, the gel was stained with Coomassie Brilliant Blue R250. Densitometric analysis of the SDS–PAGE gel was performed using TotalLab software (Nonlinear dynamics, USA).

*Determination of rhGH Concentration by ELISA*

Concentration of rhGH in the samples was measured by an enzyme-linked immunosorbent assay (ELISA) using a commercially available test kit (hGH ELISA, Roche) according to the manufacturer’s protocols. hGH standards were prepared in 1:2 dilution steps from 400 to 12.5 pg/mL. Test samples were diluted in the range of standard concentrations. Two-hundred μL of each standard and sample was added to the pre-coated wells and incubated for 1 h at 37 °C. Then the solution was removed thoroughly and the well rinsed 5 times with 250 μL of washing buffer for 30 s each. Following the washing step, 200 μL of anti-hGH-DIG was added to each well and incubated for 1 h at 37 °C. The washing step was repeated again and 200 μL of anti-hGH-POD was added to each well and incubated for 1 h at 37 °C. Two-hundred μL of POD substrate was added into each well and incubated at 20 °C until color development. The absorbance of the sample was measured at 405 nm with a reference wavelength at 490 nm, using an ELISA reader (Spectrostar Nano, BMG, Germany). The standard curve was constructed by plotting the absorbance for the hGH standards on the y-axis versus the hGH standard concentrations on the x-axis. 

*Statistical tests*

Statistical analysis was performed by GraphPad Prism 6.01 software (GraphPad software, La Jolla, USA). Data were analyzed by one-way ANOVA followed by Tukey›s post-hoc*. *Differences with *p*-values of < 0.05 were regarded as significant. 

## Results

*Cell density measurement*

**Figure 1:**
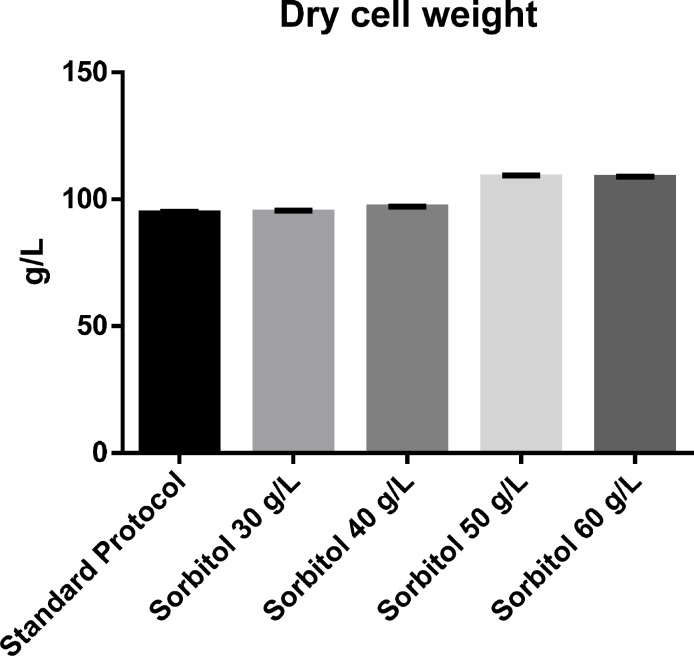
The effect of various sorbitol substrate concentration on cell density

The effect of sorbitol concentration combined with stepwise methanol addition in mixed-feed strategy on cell density was investigated during 30 h induction phase. In the methanol fed-batch strategy (basic protocol) cell density, as DCW, was 94.38 g/L. In the presence of sorbitol, cell density was found to be 94.73, 96.83, 108.5 and 108.38 g/L at a concentration of 30, 40, 50 and 60 g/L sorbitol respectively. The effect of sorbitol substrate concentration on cell density was shown in Figure 1.

It has been reported that adding sorbitol to the medium enhances biomass production, achieving higher recombinant protein (20-25, 27 and 28). By increasing the concentration of sorbitol to 50% at the beginning of methanol induction phase (MIP), a significant increase in cell density was observed. Since, there was no significant difference between sorbitol co-substrate at concentrations of 50 g/L and 60 g/L, so, in terms of cell density a mixed feed containing 50 g/L sorbitol was considered as the optimal concentration. 

**Figure 2:**
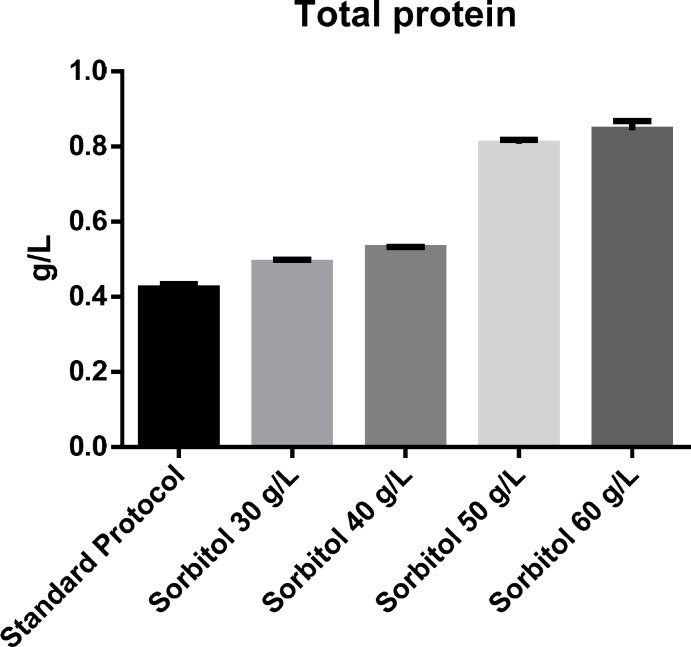
The effect of different concentrations of sorbitol on total protein determined using Bradford protein assay.

**Figure 3:**
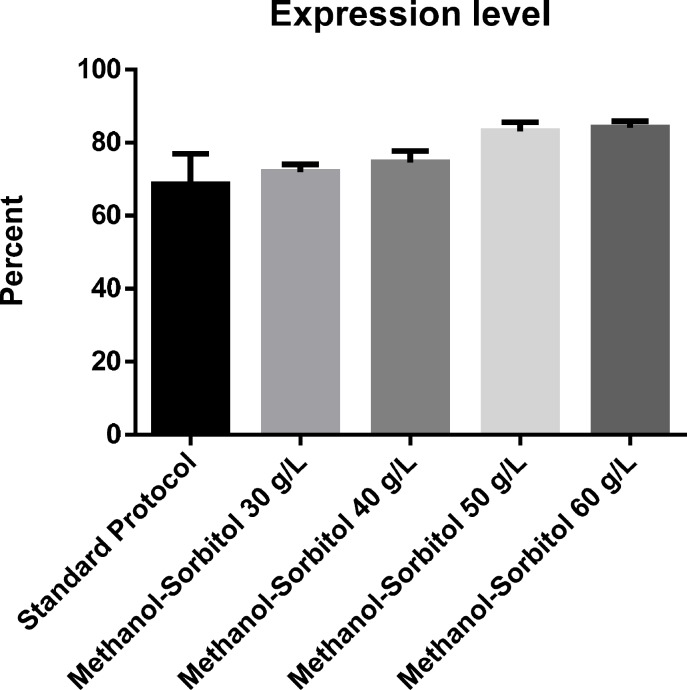
The effect of various concentration of sorbitol on the level of protein expression (percent

**Figure 4:**
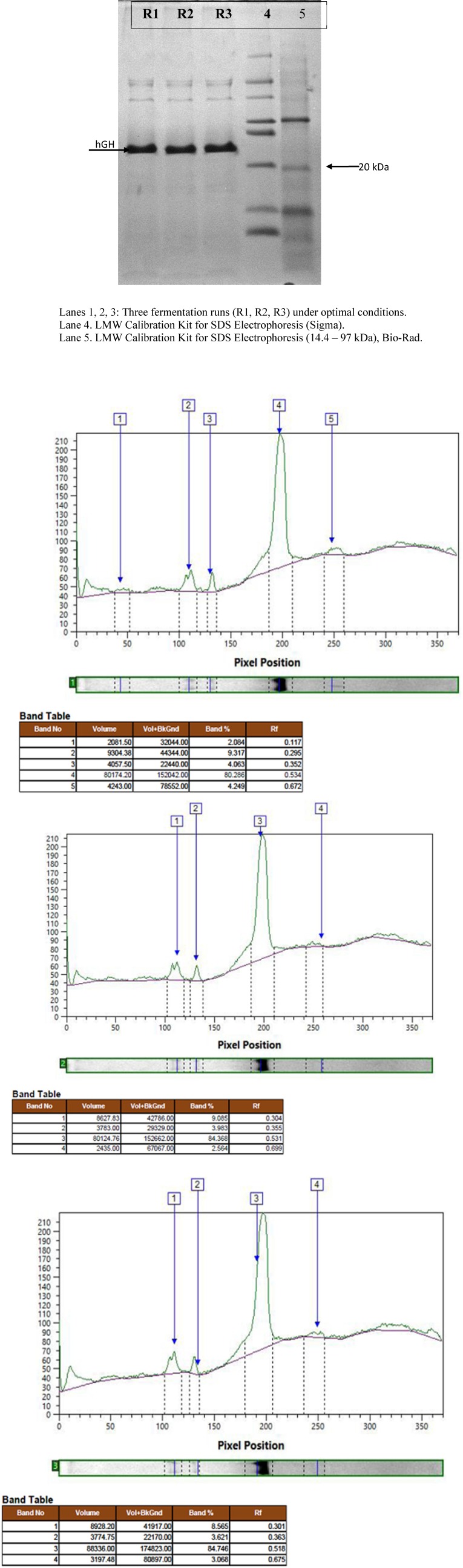
Protein expression and densitometric analysis of three fermentation runs at concentration of 50 g/L

**Figure 5:**
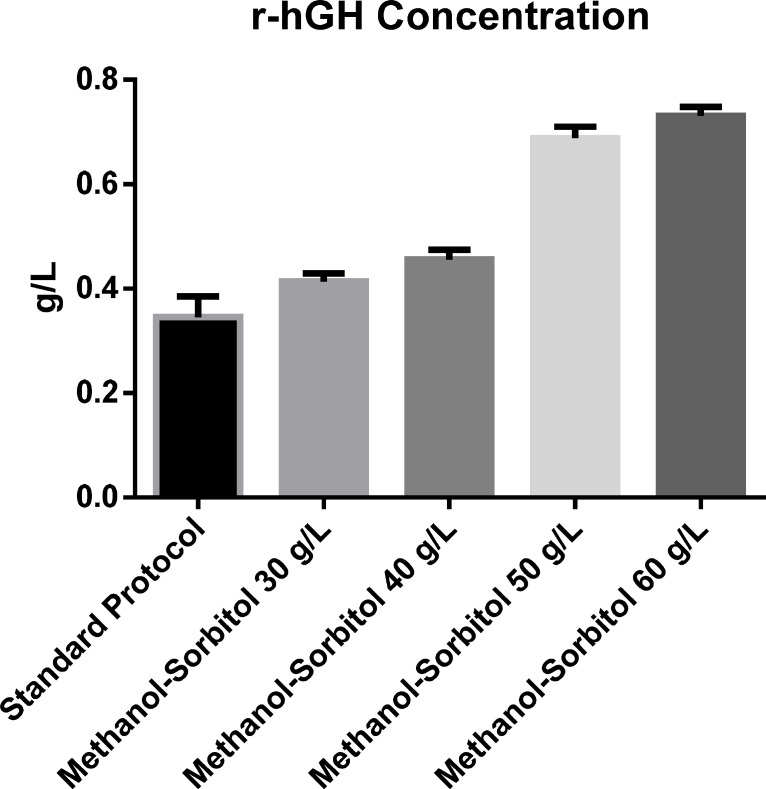
The effect of sorbitol concentration on rhGH production

Furthermore, dry cell weight was calculated using the afore-mentioned equation. Results obtained from comparison of different concentrations of sorbitol in mixed feed strategy indicate that there is an increase in dry cell weight with an increase in sorbitol concentration. However, the maximum dry cell weight was observed in the presence of 50 g/L of sorbitol and, again, beyond this concentration no significance increase was observed.

*Determination of total protein*

The effect of sorbitol-methanol mixed feeding strategy on total protein concentration was also investigated. Addition of different concentrations of sorbitol resulted in significant (*p*-value = 0.05) increase in total protein concentration in culture medium. According to the obtained results total protein concentration at 30, 40, 50 and 60 g/L of sorbitol was 0.423, 0.490, 0.530, 0.807, 0.844 mg/mL respectively. Statistical analysis using ANOVA revealed a significant difference (*p-*value = 0.05) between sorbitol mixed-feed at concentrations of 30, 40 and 50 g/L on total protein concentration. However, 50 g/L sorbitol co-substrate was found to be the optimal concentration for induction, as increasing the concentration of sorbitol beyond that did not lead to a significant increase in protein concentration. Total protein concentration under optimal condition reached 0.844 mg/mL which was approximately 2-folds higher than the concentration obtained with the basic protocol (Figure 2).

*Expression level*

The effect of induction with different concentrations of sorbitol on rhGH expression level showed a similar pattern of increase to that of total protein (Figure 2). However, although at 30 and 40 g/L concentrations of sorbitol, an increase in the expression level was seen, the difference with the basic protocol was not significant, partly due to the high standard deviation of the results of the basic protocol. However, at 50 g/L concentration of sorbitol, the increase in the expression level was large enough to be significantly higher than the basic protocol about 1.2-folds. Again, no significant difference was found between the result of sorbitol concentration of 50 and 60 g/L (Figure 3). The expression level under optimal conditions was 82.66%. Figure 4 shows protein expression and densitometric analysis of three fermentation runs at concentration of 50 g/L.

*Measurement of rhGH production*

The effect of sorbitol concentration in mixed feed strategy with stepwise addition of methanol on the production of rhGH is shown in Figure 5. The rhGH concentration in the basic protocol was 0.345 g/L but the amount was increased about 2-fold when concentration of sorbitol used was 50 g/L. Statistical analysis of different concentrations of rhGH secreted into the culture medium at different concentrations of sorbitol, in comparison with the basic protocol, showed significant difference in rhGH concentration produced. However, similar to the above results, no statistical significant difference was observed between the effect of sorbitol concentrations of 50 and 60 g/L. So, once again, 50 g/L was found to be the optimal amount of sorbitol with an rhGH production of 0.688 g/L.

## Discussion

The feeding rate in MIP is one of the key factors for maximizing protein production (33, 34), because protein production is directly or indirectly associated with cell growth (35). So, different approaches have been proposed to optimize MIP. One strategy to increase the productivity of *Pichia *expression is the use of a multicarbon substrate in addition to methanol. Accordingly, mixed feeding strategies have been studied to partially replace or at least complement methanol with other carbon sources such as glycerol (14-16) glucose (17-19) or sorbitol (20-25). Among these, sorbitol as a non-repressive carbon source was found the most promising one (27). The benefits of methanol/sorbitol co-feeding with various strategies have been reported by other researchers (20-25, 28 and 29). In the mixed-feed strategy reported by Celik *et al.* the highest rhGH production and cell concentration achieved was 0.64 g/L and 105 g/L, respectively. In the strategy used, methanol was fed to the system at a predetermined rate of 0.03/h and sorbitol concentration was kept at 50 g/L at t = 0-15 h of rhGH production phase. These results were obtained following 42 h induction time (28).

In the present study, not only the effect of different concentrations of sorbitol was examined but also induction was performed with a continuous feed containing increasing concentration of methanol. The results obtained indicated that addition of sorbitol at a concentration of 50 g/L at the beginning of the induction phase and continuing the induction with methanol for 30 h resulted in maximum biomass and rhGH production. Under these optimal conditions the cell biomass achieved was 108 g/L (DCW), total protein 0.807 g/L, rhGH expression level 83.1% and rhGH concentration was 0.688 g/L. These results were obtained following an induction time of 30 h which is considerably shorter in comparison to the previous studies.

Moreover, the productivity of mixed-feed strategy at optimal conditions within 24 h was comparable with 30 h induction with the basic protocol.

The shorter induction phase required by the strategy developed in this study has advantages other than simply lower costs. It was reported that during fermentative production of extracellular recombinant proteins, the product was degraded increasingly after 48 h of induction and the rate of degradation increased towards 72 h (36). So, by using shorter induction times, the risk of degradation of the recombinant protein could be reduced and hence product yield may improve. Furthermore the longer the induction time, the higher the amount, and even possibly the variety, of the impurities produced. On the other hand, the control of the product-related impurities during the upstream processes by using a shorter induction phase, leads to an increase in the efficiency of purification processes, and/or reduction of purification steps required, to remove such impurities to an acceptable level in the downstream stage. It is worth noting that in this study heat production and oxygen consumption rates was reduced during the induction phase without affecting rhGH production. This leads to reduced production costs especially in large scale manufacturing processes.

## References

[1] Ahmad M, Hirz M, Pichler H and Schwab H (2014). Protein expression in Pichiapastoris: recent achievements and perspectives for heterologous protein production. Appl. Microbiol. Biotechnol..

[2] Kamarthapu V, Ragampeta S, Rao KV, Reddy VD (2013). Engineered Pichiapastoris for enhanced production of S-adenosylmethionine. AMB Express.

[3] Ramón A, Marín M (2011). Advances in the production of membrane proteins in Pichia pastoris. Biotechnol. J..

[4] Liu WC, Gong T, Wang QH, Liang X, Chen JJ, Zhu P (2016). Scaling-up fermentation of Pichia pastoris to demonstration-scale using new methanol-feeding strategy and increased air pressure instead of pure oxygen supplement. Sci. Rep..

[5] Weidner M1, Taupp M, Hallam SJ (2010). Expression of recombinant proteins in the methylotrophic yeast Pichia pastoris. J. Vis. Exp..

[6] Stratton J, Chiruvolu V, Meagher M, Higgins DR, Cregg JM (1998). High cell-density fermentation. Methods in Molecular Biology: Pichia Protocols.

[7] Cos O, Ramón R, Montesinos JL, Valero F (2006). Operational strategies, monitoring and control of heterologous protein production in the methylotrophic yeast Pichia pastoris under different promoters: a review. Microb. Cell Fact..

[8] Tschopp JF, Brust PF, Cregg JM, Stillman CA, Gingeras TR (1987). Expression of the Lacz gene from 2 methanol-regulated promoters in Pichia-Pastoris. Nucleic Acid Res..

[9] Chiruvolu V, Cregg JM and Meagher MM (1997). Recombinant protein production in an alcohol oxidase-defective strain of Pichia pastoris in fed-batch fermentations. Enzyme Microb. Technol..

[10] Invitrogen Co (US) (2002). Pichia Fermentation Process Guidelines.

[11] Zhang W, Inan M, Meagher MM, Cregg JM (2007). Rational design and optimization of fed-batch and continuous fermentations. Methods in Molecular Biology: Pichia Protocols.

[12] Meyer HP, Brass J, Jungo C, Kelin J, Wenger J, Mommers R (2008). An emerging star for therapeutic and catalytic protein production. BioProcess Int..

[13] Celik E, Calık P, Oliver SG (2009). Fed-batch methanol feeding strategy for recombinant protein production by Pichia pastoris in the presence of co-substrate sorbitol. Yeast.

[14] Zhang W, Hywood Potter KJ, Plantz BA, Schlegel VL, Smith LA, Meagher MM (2003). Pichia pastoris fermentation with mixed-feeds of glycerol and methanol: Growth kinetics and production improvement. J. Ind. Microbiol. Biotechnol..

[15] Jungo C, Marison I, von Stockar U (2007). Mixed feeds of glycerol and methanol can improve the performance of Pichia pastoris cultures: A quantitative study based on concentration gradients in transient continuous cultures. J. Biotechnol..

[16] Zalai D, Dietzsch C, Herwig C, Spadiut O (2012). A dynamic fed batch strategy for a Pichia pastoris mixed feed system to increase process understanding. Biotechnol. Prog..

[17] Paulova L, Hyka P, Branska B, Melzoch K, Kovar K (2012). Use of a mixture of glucose and methanol as substrates for the production of recombinant trypsinogen in continuous cultures with Pichia pastoris Mut+. J. Biotechnol..

[18] Jorda J, Jouhten P, Camara E, Maaheimo H, Albiol J, Ferrer P (2012). Metabolic flux profiling of recombinant protein secreting Pichia pastoris growing on glucose: Methanol mixtures. Microb. Cell Fact..

[19] Inan M, Meagher MM (2001). Non-repressing carbon sources for alcohol oxidase (AOX1) promoter of Pichia pastoris. J. Biosci. Bioeng..

[20] Ramon R, Ferrer P, Valero F (2007). Sorbitol co-feeding reduces metabolic burden caused by the overexpression of a Rhizopus oryzae lipase in Pichia pastoris. J. Biotechnol..

[21] Wang Z, Wang Y, Zhang D, Li J, Hua Z, Du G, Chen J (2010). Enhancement of cell viability and alkaline polygalacturonate lyase production by sorbitol cofeeding with methanol in Pichia pastoris fermentation. Bioresour. Technol..

[22] Zhu T, You L, Gong F, Xie M, Xue Y, Li Y, Ma Y (2011). Combinatorial strategy of sorbitol feeding and low-temperature induction leads to high-level production of alkaline beta-mannanase in Pichia pastoris. Enzyme Microb. Technol..

[23] Gao MJ, Li Z, Yu RS, Wu JR, Zheng ZY, Shi ZP, Zhan XB, Lin CC (2012). Methanol/ sorbitol co-feeding induction enhanced porcine interferon-alpha production by P pastoris associated with energy metabolism shift. Bioprocess. Biosyst. Eng..

[24] Niu H, Jost L, Pirlot N, Sassi H, Daukandt M, Rodriguez C, Fickers P (2013). A quantitative study of methanol/sorbitol co-feeding process of a Pichia pastoris Mut+ /pAOX1-lacZ strain. Microb. Cell Fact..

[25] Sreekrishna K, Flickinger MC (2013). Gene expression in pichia and other methylotroph yeast. Upstream Industrial Biotechnology.

[26] Jungo C, Schenk J, Pasquier M, Marison IW, von Stockar U (2007). A quantitative analysis of the benefits of mixed feeds of sorbitol and methanol for the production of recombinant avidin with Pichia pastoris. J. Biotechnol..

[27] Gunes H, Boy E, Ata O, Zerze GH, Calıka P, Ozdamarc TH (2016). Methanol feeding strategy design enhances recombinant human growth hormone production by Pichia pastoris. J Chem. Technol. Biotechnol..

[28] Calık P, Bozkurt B, Zerze GH, İnankur B, Bayraktar E, Boy E, Orman M A, Açık E, Özdamar TH (2013). Effect of co-substrate sorbitol different feeding strategies on human growth hormone production by recombinant Pichia pastoris. J. Chem. Technol. Biotechnol..

[29] Huang J, Barent R, Inan M, Gouthro M, Roxas PV, Smith LA, Meagher MM, Cregg JM (2007). Purification of the N- and C-Terminal subdomains of recombinant heavy chain fragment C of botulinum neurotoxin serotype C. Methods in Molecular Biology: Pichia Protocols.

[30] Lee CY, Lee SJ, Jung KH, Katoh S, Lee EK (2003). High dissolved oxygen tension enhances heterologous protein expression by recombinant Pichia pastoris. Process Biochem..

[31] Bradford MM (1976). A rapid and sensitive method for the quantitation of microgram quantities of protein utilizing the principle protein dye binding. Anal. Biochem..

[32] Laemmli UK (1970). Cleavage of structural proteins during the assembly of the head of bacteriophage T4. Nature.

[33] Zhang W, Bevins MA, Plantz BA, Smith LA, Meagher M (2000). Modeling Pichia pastoris growth on methanol and optimizing the production of a recombinant protein, the heavy-chain fragment C of botulinum neurotoxin, serotype A. Biotechnol. Bioeng..

[34] Zhang W, Inan M, Meagher MM (2000). Fermentation strategies for recombinant protein expression in the methylotrophic yeast Pichia pastoris. Biotechnol. Bioprocess Eng..

[35] Sreekrishna K, Flickinger MC (2013). Gene expression in pichia and other methylotroph yeast. Upstream Industrial Biotechnology.

[36] Sinha J, Plantz BA, Inan M, Meagher MM (2005). Causes of proteolytic degradation of secreted recombinant proteins produced in methylotrophic yeast Pichia pastoris: Case Study with Recombinant Ovine Interferon tau. Biotechnol. Bioeng..

